# Genome Sequences of Poaceae-Associated Gemycircularviruses from the Pacific Ocean Island of Tonga

**DOI:** 10.1128/genomeA.01144-15

**Published:** 2015-10-15

**Authors:** Maketalena F. Male, Viliami Kami, Simona Kraberger, Arvind Varsani

**Affiliations:** a Biomolecular Interaction Centre and School of Biological Sciences, University of Canterbury, Christchurch, New Zealand; b Vuna Road, Nuku’Alofa, Tonga; c Department of Clinical Laboratory Sciences, Structural Biology Research Unit, Division of Medical Biochemistry, University of Cape Town, Observatory, South Africa; d Department of Plant Pathology and Emerging Pathogens Institute, University of Florida, Gainesville, Florida, USA

## Abstract

We sampled and analyzed 43 Poaceae plants from the Pacific Ocean island of Tonga for the presence of circular DNA viruses. From these samples, we recovered three gemycircularvirus genomes, which share >99% identity, from *Brachiaria deflexa* (*n* = 2) and sugarcane (*n* = 1). These genomes share <61% genome-wide identity with other gemycircularvirus sequences in public databases.

## GENOME ANNOUNCEMENT

A variety of novel circular replication-associated proteins (Rep) encoding single-stranded (CRESS) DNA viruses have been identified (1, 2) in dragonflies from the Pacific Ocean island of Tonga over the last few years. In an attempt to identify CRESS DNA viruses associated with plants, we collected 43 Poaceae leaf samples (16 wild grasses and 27 sugarcane) in the Kingdom of Tonga in 2014.

Approximate 1 cm^2^ of each leaf sample was used to isolate viral DNA, and circular DNA was enriched using rolling circle amplification, as described in Kraberger et al. (3). The enriched DNA was grouped and sequenced at Beijing Genomics Institute (Hong Kong) on an Illumina HiSeq 2000 sequencer (Illumina, USA). The paired-end reads were *de novo* assembled using ABySS 1.3.5 (4) (*k*-mer = 64), and contigs >500 nucleotides (nt) were analyzed using BLASTx (5) against a viral protein database. We identified three unique Rep-coding contigs, for which we designed abutting primers (GmV-1F [5′-GCGTTTTCACGGTGGAGCGAGAG-3′], GmV-1R [5′-GTCGCTACAAGAGGATATACCGCAGAC-3′], CM-1F [5′-CACTTCTGCTTAGTGGCATCGCCAC-3′], CM-1R [5′-GAAGGGCATTATTGATGCGGCCAGAC-3′], CM-2F [5′-GAGGCCGGGGCAATAAGAGAAAATGGC-3′], and CM-2R [5′-CCGCGACATTTTGTT CTTCTGGCAGTG-3′]) to screen and recover the complete sequences of these molecules from the 43 Poaceae samples, using Kapa HiFi DNA polymerase (Kapa Biosystems, USA). We recovered three circular molecules of ~2.2 kb from two *Brachiaria deflexa* samples and a sugarcane sample using the primer pair GmV-1F/R. Additionally, using the primer pairs CM-1F/R and CM-2F/R, we recovered two circular DNA molecules of ~1.1 kb from the same *B. deflexa* samples in which we recovered the ~2.2-kb genomes. All PCR amplicons were cloned into pJET1.2 plasmid (Thermo Fisher Scientific, USA) and Sanger sequenced at Macrogen, Inc. (South Korea).

The three 2,253-kb genomes from two *B. deflexa* samples and a sugarcane sample share >99% pairwise identity and are most similar to gemycircularviruses, sharing <61% genome-wide identity. Their Rep and capsid protein (CP) share <54% and <40% identity, respectively, with those of other gemycircularviruses. Based on this, we tentatively named them Poaceae-associated gemycircularvirus-1 (PaGmV-1) (isolates STO14, STO15, and STO18). Gemycircularviruses are circular single-stranded DNA viruses with ~2-kb ambisense genomes encoding a CP on the virion sense and a Rep on the complementary sense. Gemycircularvirus genomes have been recovered from a wide variety of samples, including insects, fecal matter, treated sewage, river sediments, human and animal serum, plants, and fungi (1, 3, 6–16). The gemycircularvirus with a known host is *Sclerotinia sclerotiorum* hypovirulence-associated DNA virus-1 (i.e., *S. sclerotiorum*); however, it is likely that other gemycircularviruses also infect fungi, since gemycircularvirus Rep-like sequences have been identified in various fungal genomes (17).

The two 1,162-nt and 1,127-nt circular molecules recovered using the primer pairs for CM-1F/R (tentatively named *B. deflexa*-associated circular DNA molecule-1 [BdaCM-1], with isolates STO14 and STO15) and CM-2F/R (tentatively named BdaCM-2, with isolates STO14 and STO15) each share >99% pairwise identity and only encode Reps that appear to have introns (~100 to 200 nt). BdaCM-1 and -2 share ~67% identity, and their spliced Reps share <40% identity with Reps of other CRESS DNA viruses whose sequences are available in public databases.

This is the first report of single-stranded DNA (ssDNA) viruses and circular DNA molecules associated with wild grasses and sugarcane in the Kingdom of Tonga.

### Nucleotide sequence accession numbers.

The complete genome sequences of PaGmV-1 (isolates STO14, STO15, and STO18), BdaCM-1 (isolates STO14-10889 and STO15-10889), and BdaCM-2 (STO14 and STO15) have been deposited at GenBank under accession numbers KT253577 to KT253583.
